# Drosophila Larval NMJ Dissection

**DOI:** 10.3791/1107

**Published:** 2009-02-04

**Authors:** Jonathan R. Brent, Kristen M. Werner, Brian D. McCabe

**Affiliations:** Department of Physiology and Cellular Biophysics, Columbia University College of Physicians and Surgeons

## Abstract

The *Drosophila* neuromuscular junction (NMJ) is an established model system used for the study of synaptic development and plasticity. The widespread use of the *Drosophila* motor system is due to its high accessibility. It can be analyzed with single-cell resolution. There are 30 muscles per hemisegment whose arrangement within the peripheral body wall are known. A total of 35 motor neurons attach to these muscles in a pattern that has high fidelity. Using molecular biology and genetics, one can create transgenic animals or mutants. Then, one can study the developmental consequences on the morphology and function of the NMJ. In order to access the NMJ for study, it is necessary to carefully dissect each larva. In this article we demonstrate how to properly dissect *Drosophila* larvae for study of the NMJ by removing all internal organs while leaving the body wall intact. This technique is suitable to prepare larvae for imaging, immunohistochemistry, or electrophysiology.

**Figure:**
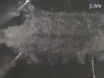


## Protocol

### Before you start

1. All cultures should be grown at 25° C.
2. HL3.1 dissection buffer may be prepared in advance.  Take a 50 ml aliquot of HL3.1 and keep it on ice.
3. Prepare 15 ml of fresh 3.5% Formaldehyde in HL3.1. Keep it on ice.

### Larval Dissection

1. Put a drop of cold HL3.1 on the dissection plate. This will keep the animal from drying and stun the animal making it easier to work with.
2. Using the **long forceps**, select a wandering third instar larva and place it in the drop of HL3.1.
3. First, use **short forceps** to grasp a **minutien pin**. Place the pin between the posterior spiracles. The animal will usually try to crawl away from the pin stretching itself out and making it easier for you to place a pin squarely in the head of the larva near the mouth hooks. Stretch the animal out lengthwise. This will help you maximize the amount of exposed body wall you can achieve during cutting.  Note : If you choose to  pin the head first, the animal may wrap its body around the pin or whip its body back and forth making it difficult to  pin. You can usually counter this by removing the HL3.1 and putting a fresh cold drop stunning the animal again. The ambidextrous scientist can also just grab the animal and hold it in place.
4. Using spring scissors make a horizontal incision just anterior to the posterior pin on the dorsal side of the larva. Place one blade of the scissors into the incision and make a vertical cut along the dorsal midline toward the rostral end of the larva. At the rostrum of the animal make horizontal incisions to the left and right of the pin. Note: the finished result should look like an I on the dorsal side of the larva along the rostrocaudal axis.
5. Remove the organs. Begin by putting several additional drops of HL3.1 on the larva. This will enable the organs to float up out of the body allowing you to remove them much more easily. First, remove the tracheal system. Second, use the forceps to grab the rest of the organs and remove them.
6. During step 3 you made a left flap and right flap in the body wall. Pin the flaps in a clockwise order making sure to stretch the body wall both horizontally and vertically.

### Fixation

Fix each animal in 3.5% formaldehyde in HL3.1 for 25 minutes. Wash the animal twice in HL3.1 for 5 minutes.

### Storage

Remove the pins and transfer the larvae to a 1.5 ml tube containing 1X PBS. If you plan to image the NMJ using fused fluorescent tags, you should image the animals within two days.  You may store the dissected larvae for up to one week at 4°C.

### Representative results

There are several key components to a properly dissected *Drosophila* larva. First, one must take extra care not to damage the muscles, especially muscles 4, 6, and 7.  These are the most popular muscles to study and if they are damaged discard the fillet prep and dissect another animal. Second, one must make sure that the animal is maximally stretched so that each muscle and NMJ can be distinguished.

## Discussion

The dissection technique demonstrated in this video can be used to prepare *Drosophila* larvae for a variety of experimental techniques. If fluorescent protein tags are present, the larvae can be mounted and imaged immediately. Otherwise, immunostaining can be performed in order to mark specific synaptic compartments. In addition, electrophysiology can easily be performed on the dissected larvae to evaluate the functioning of neurotransmission.

The NMJ of *Drosophila* has gained great popularity since the early studies that illuminated its basic structure and function.^1-4^ Many of the molecules that have been identified in studies of the development of the *Drosophila* NMJ are conserved in vertebrates.^4^ Therefore, many of the insights learned through studies of the *Drosophila* NMJ are applicable to synaptic biology in all living beings. Some possible applications include the study of molecules involved in axon guidance, motor neuron disease, learning, and memory.
